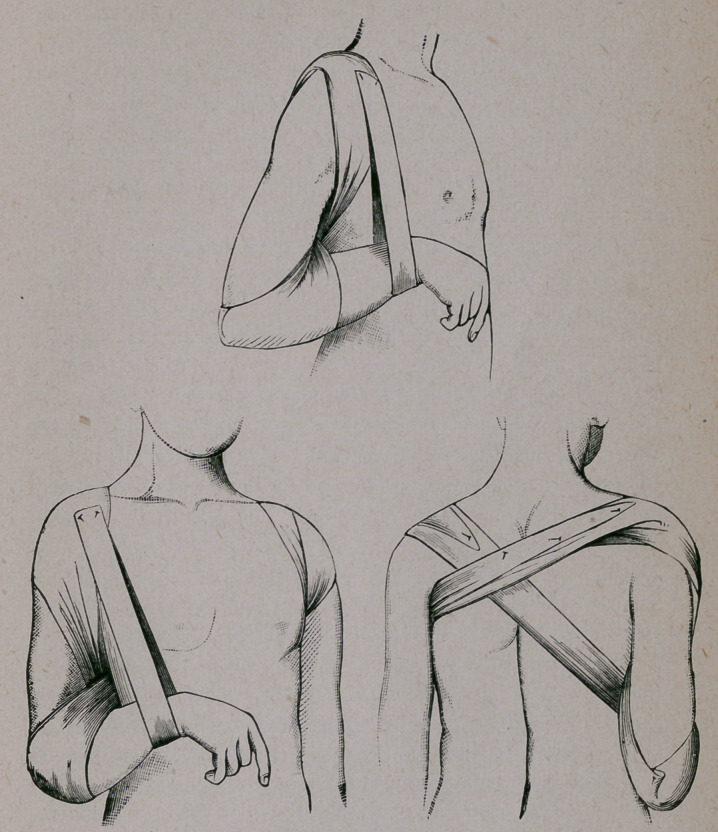# The Surgery of the Upper Extremities

**Published:** 1889-02

**Authors:** Edward M. Moore

**Affiliations:** Rochester, N. Y.


Buffalo Medical and Surgical Journal

Vol. XXVIII. FEBRUARY, 1889. No. 7

### Original Communications.

### THE SURGERY OF THE UPPER EXTREMITIES.

### Part I.

### TREATMENT OF THE CLAVICLE WHEN FRACTURED OR DISLOCATED.[1]

By EDWARD M. MOORE, M. D., Rochester, N. Y.

The symptoms of fractured clavicle are very constant. The shoulder goes downward, inward, and forward, in obedience to the action of gravity, the shape of the thorax and the tonic power of the pectoral muscles; and, as a sequence of this altered position, the *inner* fragment almost surely rides the outer one. It is of this common condition only that I speak.

It is well known that the treatment of this fracture is conducted generally upon two plans, with a great variety of modifications of detail. One is by the use of the axillary pad, which is the fulcrum for the humeral lever; the other is by the figure eight (8) bandage, which draws the shoulders backward by a band passing around the axilla. There are other plans, but these prevail.

*The tension of the clavicular fibres is the chief indication for rectifying the deformity. There are other considerations, however, among the principal of which is the position of the scapula.* When we place a patient in a chair, and, standing behind him, seize the upper part of the arms near the shoulder and draw them backward, the replacement of the fractured ends becomes perfect, almost with certainty. But when, with more force, we use the axillary pad or figure eight (8) bandage, the restoration is so

---

1. This paper forms a part of the subjects of an address before the Central New York Medical Association, November 20, 1888.

rare that many surgeons assert its impossibility. I criticise the axillary pad, because it ensures the relaxation of the clavicular fibres of the great pectoral. If this results from its action, we lose the antagonism of the sterno-cleido-mastoid, and therefore, no matter how high we elevate the shoulder, the end of the inner fragment will be the higher. But, if the shoulder is carried backward, and the clavicular fibres of the pectoral muscle are rendered tense, the riding inner fragment will be brought down, thus securing perfect apposition. This results from the well-known treatment by recumbent posture, and from the attitude in the chair, as above described.

The attachment of the great pectoral to the humerus, it will be remembered, is quite peculiar. The muscle makes a half turn upon itself, and is inserted for a full inch and a half along the bicipital groove, the thoracic fibres running to its upper, and the clavicular to its lower part. It must be obvious that the tension of the fibres in the same muscle will vary according to the position of the humerus. If the arm is carried backward, it moves from the shoulder joint as the fulcrum, the lower portion of the muscular insertion describing the larger circle, thus involving a greater tension of the clavicular fibres. By this tension we antagonize the sterno-cleido-mastoid muscle, and if the shoulder be carried back far enough, the apposition will be perfect, provided no inequality of the fractured surfaces prevents, which will often happen in oblique fractures, even when the length is fully obtained.

Before presenting a plan of treatment, I will criticise, in the first place, the axillary pad. As the large end of this pad is placed upwards, it operates, of course, upon the humerus just at the border of the axilla. Its rationale is that of a fulcrum for the humerus as a lever. The motion on the fulcrum, if a mathematical line, is *nil*. The pad is opposite the attachment of the clavicular fibres of the pectoral muscle. At the point of their insertion there is no motion, no matter how much force we may use. The shoulder is thrown out as far as the thoracic fibres of the pectoral muscle will allow. The upper end of the bicipital groove is the point of insertion for these fibres, which are put upon the stretch, because it is removed from the fulcrum. The clavicular fibres of the pectoral are relaxed and kept so, and the sterno-cleido-mastoid draws up the inner fragment.

I also criticise the figure eight (8) bandage for producing a similar relaxation of the clavicular fibres of the pectoral muscle. This bandage acts upon the lower border of the muscle, which is composed entirely of thoracic fibres, and the more tightly it is drawn, the more will it curve its axillary border, and incidentally shorten it. The fibres of the clavicular portion are not bent, and of course, not shortened. Thus they are inevitably relaxed by the drawing in of the humerus. It will, therefore, be seen that, in the effort to carry the shoulder out by the axillary pad, or backward by the figure eight (8) bandage, the condition of tension of the thoracic, and relaxation of the clavicular fibres of the pectoral muscle results. The indication, therefore, to be fulfilled, is to reverse the action, and, by carrying the humerus backward, make tense the clavicular and relax the thoracic fibres of the pectoralis major muscle. This motion is at the shoulder-joint as a pivot. The tension of the pectoral fibres is now reversed—the lower portion of the bicipital groove is moved through a greater circle than the upper, and the clavicular fibres are rendered tense in proportion to the distance the humerus is carried back.

*No plan that leaves the humerus perpendicular can make the clavicular fibres tense.*

The scapula must also be moved as near the spine as can be borne, for the obvious reason that the thorax is the most salient at this part, and a sliding of the scapula thus produces a movement of the shoulder backward and outward. As it goes back it rises. I also make criticism upon the effect of the shawl, Fox's or Dessault's bandage, in their operation on the scapula. If the hand be placed upon the inferior angle of the scapula and the shoulder raised, keeping the humerus perpendicular, it will be seen that the scapula moves forward upon the thorax at its lower border. This criticism, however, does not obtain with reference to the figure eight (8) bandage.

This is in the wrong direction, and although it is the lower angle, it nevertheless allows the whole scapula to come forward, and thus shorten the broken clavicle.

The obvious indications in the treatment of this fracture, therefore, consist in the use of any bandage or posture that will carry the humerus backward and hold it toward the side. The effect of carrying the arm backward is to move the scapula toward the spine, thus fulfilling the first important indication. But besides this, as stated above, the clavicular fibres are rendered tense by this attitude, and thus the only remaining indication is fulfilled. Some aid may, perhaps, be obtained by lifting the shoulder from the elbow, but this is subordinate.

In order to obtain the results stated I have resorted to various devices; but to treat fractures well we must be able to find our appliances in every house, and after much experimenting I use a shawl, or piece of cotton cloth, which, when folded like a cravat, eight inches in breadth at the center, should be about two yards long. Placing this at the center across the palm of the surgeon, he seizes with his hand the elbow of the patient, which corresponds with the broken clavicle. The two ends of the bandage hang to the floor. The one falling inward toward the patient is carried upward, in front of the shoulder and over the back, making a spiral movement in front of the shoulder. This is intrusted to an assistant. The outer end is then carried across the forearm, behind the back, over the opposite shoulder and around the axilla. This meets the other end, which may be carried under the axilla and over the shoulder of the opposite side, thus making the figure eight (8) turn around the sound shoulder. This twist, it will be seen, makes also the figure eight (8) turn around the elbow of the affected side. I therefore style the bandage:

"THE ELBOW FIGURE EIGHT (8.)"

The forearm should be sustained by a sling which raises it to an acute angle, in order that gravity may assist in moving the whole arm backward. This is best done by a simple strip three or four inches wide, which may be pinned to the shoulder bandage. Any tendency on the part of the shawl to slide from the shoulder may be arrested by a pin thrust at the crossing.[1] The bandage at the elbow is kept in place by folding the upper part that fits the arm, and securing it by a pin. This makes a sort of cup for the elbow.

The bandage is worn with ease, except on the sound shoulder, which is vexed by the pressure of the ordinary figure eight (8).

1. These figures illustrate the application of the dressing, without descriptive titles.

It will be observed that the shawl, as it passes in front of the arm, does not press on the axillary border of the pectoral muscle, and, therefore, avoids the objection of the ordinary figure eight (8). As it passes over the forearm, and over the opposite shoulder, it lifts up the arm like the ordinary shawl bandage.

Additional evidence of the correctness of the views herein stated is to be found in the fact that dislocations of the clavicle are almost surely retained by it while the patient keeps the erect posture. This, I think, cannot be said of any other plan. Nothing but absolutely full extension can retain the luxated surfaces at either end of the clavicle.

It has been objected to the use of the bandage that the pressure on the axilla of the unaffected side becomes intolerable as it passes under the belly of the great pectoral. This may be modified by passing it through a rubber tube an inch, or even less, in diameter. Pressure, even of a severe kind, can be borne if there is some relief from it at frequent intervals. This can be obtained by taking the recumbent posture and then carrying the arm of the unaffected side upward. This posture secures the apposition of the bones without a bandage, as has always been known. It is an easy relief, and can be used very frequently through every day. Another method is to rest the forearm on the arm of a chair while sitting, and lean forwards. The shoulder is carried backwards, and the bandage is rendered slack at all points.

When the arm is spare, and the bones stand out, pressure at the elbow of the affected side is also often uncomfortable. This can be relieved by padding alongside the tender point, and is best governed by the feelings of the patient, who, by frequent change of pads, can obviate this almost entirely. If the end of the olecranon is the sore point, a round hole cut in the bandage will generally afford complete relief.

To secure a proper retention, the patient should be seen every day for a week, and the bandage tightened a little. This is necessary to get rid of the stretching of the cloth. After this time it will not yield, but, of course, the patient must be seen at intervals of a few days. The roller that is used as a sling must be adapted to every case, for such is the difference of shape of each person, that it will slip forwards or backwards at times. Sometimes it is the best method to pin one end in front and the other behind the shoulder to the bandage.

A question may be raised as to what may be considered a perfect cure of a broken bone. Of course, there are cases where the irregularities of the broken surfaces can be brought so as to inosculate, but it is very certain that such conditions are extremely rare. We have to regard the cure of a fracture as perfect when the full length of the bone is attained and the line of the shaft is true. Projecting points become absorbed, spaces are filled up, and the process of absorption finally reduces the local enlargement, and the cure should now be regarded as perfect. All this can be attained in most cases of fractured clavicle by this method, if care is used.

As has been stated above, the fact that the luxations are perfectly restored by the method, proves the fact of an entire extension of the bone to its full length. A shortening of the eighth of an inch would displace a luxation. I have now nineteen reports of success in the treatment of the luxations, gathered from the practice of physicians over a wide extent of territory.

## Figures and Tables

**Figure f1:**